# 1206. A Quality Improvement Project Utilizing Clinical Decision Support (CDS) to Optimize Outpatient Antibiotic Prescribing

**DOI:** 10.1093/ofid/ofad500.1046

**Published:** 2023-11-27

**Authors:** Matt Mason, Craig Shapiro, Shannon Chan

**Affiliations:** Nemours Children's Hospital, Delaware, Wilmington, Delaware; Nemours Children's Hospital, Delaware, Wilmington, Delaware; Nemours Children's Hospital, Delaware, Wilmington, Delaware

## Abstract

**Background:**

Antimicrobial resistance remains one of the greatest threats to public health. A major contributing factor is overuse of antibiotics, with most prescribing occurring in the outpatient setting. CDS in the outpatient setting can improve antibiotic use by optimizing durations of therapy. The primary objective of this study was to assess the impact of CDS on prescribing of amoxicillin (AMX) and amoxicillin-clavulanate (AMC) for treatment of acute otitis media (AOM). The secondary objective was to assess prescribers’ perception of the CDS.

**Methods:**

The Nemours Children’s Health Antimicrobial Stewardship Program (ASP) developed antibiotic order panels (OPs) available for ambulatory care providers. The OPs provided guidance for drug formulation, dose, and duration. Providers were notified of implementation through email. Prescribed durations for AMC and AMX for AOM were compared for 3 months pre- and post-implementation. A 14-question survey was emailed to primary care providers to assess perceptions of the OPs and the impact on prescribing practices.

**Results:**

The OP utilization rate for AMC from February to April 2022 was 27.7%. In patients ≥2 years, OP utilization resulted in a decreased average duration of therapy compared to no OP utilization, 7.6 vs 9.8 days, respectively. The OP utilization rate for AMX for AOM from April to June 2022 was 15% (342/2356). In patients ≥2 years old, 5-day and 7-day prescriptions increased from 1.7% (8/472) and 9.5% (45/472) to 11.4% (193/1688) and 12.3% (207/1688), respectively. There was a 40% response rate to the survey; 75% reported awareness of the OPs and 72.7% reported routine use. The OPs were most helpful in selection of correct dose (83.3%), duration of therapy (81.3%), and indication (77.1%). Most providers reported improved efficiency (77.1%) and agreed that the OPs led to shorter durations of therapy (81%).

**Conclusion:**

Development of antibiotic specific OPs with defaulted durations can be an effective strategy to improve outpatient prescribing of antibiotics. Providers are open to CDS to improve prescribing practices. Education and evidence-based support for the recommended changes in prescribing could further maximize providers’ engagement and implementation of these changes.

**Disclosures:**

**All Authors**: No reported disclosures

